# Global sorghum production dataset for temperate to subtropical regions at subnational scale over 2000–2020

**DOI:** 10.1016/j.dib.2025.111935

**Published:** 2025-07-28

**Authors:** Mohsen Davoudkhani, Nicolas Guilpart, David Makowski, Nicolas Viovy, Philippe Ciais, Ronny Lauerwald

**Affiliations:** a Université Paris-Saclay, INRAE, AgroParisTech, UMR ECOSYS, Palaiseau, France; b Université Paris-Saclay, INRAE, AgroParisTech, UMR Agronomie, Palaiseau, France; c Université Paris-Saclay, INRAE, AgroParisTech, Unit Applied mathematics and computer science, Palaiseau 91120, France; d Laboratoire des Science du Climat et de l'Environnement, CEA, CNRS, Université de Versailles – Saint Quentin en Yvelines, IPSL, 91191, Gif-sur-Yvette, France

**Keywords:** Sorghum, Yield, Harvested area, Production, Historical data

## Abstract

Sorghum is a crop of growing interest due to its heat tolerance compared to other crops and better adaptation to future hot and dry summers. The establishment of a global dataset for sorghum yields over the past two decades can support the development of sustainable agricultural practices in the face of climate change. For the first time, this study aimed to establish a global dataset for grain sorghum yields from 2000 to 2020 for temperate to subtropical regions. Data was collected from national databases of eight countries including France, Italy, Spain, Argentina, Mexico, USA, China, and Australia, covering 85 % of the total sorghum production of the world’s temperate to subtropical regions in this period. We collected data from publicly accessible national databases, with data recorded at various administrative levels: county level for USA and Argentina, municipal level for Mexico, NUTS3 level for European countries, provincial level for China, and Natural Resource Management Regions for Australia. The dataset comprises 27,222 data points of grain sorghum yield, harvested area, and production values, each obtained for one specific year and averaged over a specific administrative unit at the subnational scale. The dataset is structured by country and includes raw and processed files, along with geospatial boundaries of administrative units. The dataset can be used to develop crop models, machine learning algorithms, and statistical models for predicting sorghum yields under different climate scenarios. The dataset is also suitable for climate impact assessments, land-use studies, and policy planning in support of climate-resilient agriculture.

Specifications Table

**Table:** 

Subject	Earth & Environmental Sciences	
Specific subject area	Subnational-scale sorghum yield, production, and harvested area data for temperate-to-subtropical regions, supporting climate-resilient agriculture	
Type of data	*Table* *Figure* *Time series data* *Geospatial data*	
Data collection	We collected sorghum data from the national databases of eight countries, including France, Italy, Spain, Argentina, Mexico, the USA, China (mainland), and Australia. Data were collected over the period 2000 to 2020 for subnational administrative entities (Table 1, Figure 1). This global dataset comprises a total of 27,222 data entries. Each entry includes the harvested area, production, and yield of grain sorghum for one specific territorial unit and year.	
Data source location	Argentina	https://datosestimaciones.magyp.gob.ar/reportes.php?reporte=Estimaciones
	Australia	https://www.abs.gov.au/statistics/industry/agriculture/agricultural-commodities-australia
	China	https://data.stats.gov.cn/easyquery.htm?cn=E0103
	France	https://agreste.agriculture.gouv.fr/agreste-web/disaron/SAANR_DEVELOPPE_2/detail/
	Italy	https://www.istat.it/en/agriculture?data-and-indicators
	Mexico	https://nube.siap.gob.mx/cierreagricola/
	Spain	https://www.mapa.gob.es/es/estadistica/temas/estadisticas-agrarias/agricultura/esyrce/
	The USA	https://www.nass.usda.gov/Data_and_Statistics/
Data accessibility	Repository name: **Github** Data identification number: https://doi.org/10.5281/zenodo.15426456 Direct URL to data: https://github.com/mdavoudkhani/Global-sorghum-production-dataset-for-temperate-to-subtropical-regions-at-subnational-scale.	
Related research article	*none*	

## 1 Value of the Data

• This is the first global database of sorghum yields under temperate to subtropical climates.
• **Climate adaptation research:** This dataset provides sorghum production data at the finest available subnational administrative units across diverse temperate to subtropical regions, allowing researchers to analyse how sorghum performs under different climatic conditions and assess its potential as a climate-resilient alternative to other cereals in these regions because of its high water and nitrogen use efficiency, and ability to better withstand heat and water stress.
• **Yield modelling and prediction**: Sorghum data at the high spatial resolution (subnational administrative units) and temporal coverage (2000–2020) allows researchers to develop and validate crop models, machine learning algorithms, and statistical models for predicting sorghum yields based on environmental, climate, and management factors.
• **Comparative agricultural studies**: Researchers can use this dataset coming from eight major producing countries covering 85 % of temperate to subtropical sorghum production to implement cross-country comparisons to identify best practices, analyse productivity gaps, understand how different agricultural systems impact sorghum performance, and optimize land allocation between different crops.

## 2 Background

Sorghum is the fifth most widely cultivated cereal crop globally in terms of both grain production and cultivation area [1,2]. Sorghum is usually considered a good alternative to other cereal crops because of its high water and nitrogen use efficiency, and ability to better withstand heat and water stress [3,4], and is thus a preferred crop in environments with limited water availability [[5], [6], [7], [8]]. Moreover, sorghum is a cost-effective crop for farmers, requiring minimal input of fertilizers and pesticides. Further, its high root-to-shoot ratio contributes to enhancing soil carbon sequestration and soil physical properties [[9], [10], [11], [12]].

Here, for the first time, we present sorghum data from the national databases of eight countries, including France, Italy, Spain, Argentina, Mexico, the USA, China (mainland), and Australia. This dataset covers 40 % of the total production of the world from 2000 to 2020. Notably, our database includes data from temperate to subtropical regions, excluding tropical sorghum-producing nations such as those in Africa, India, Brazil, and Bolivia. This choice enabled us to cover approximately 85 % of sorghum production in these specific climatic zones during the same period to represent sorghum cultivation trends in temperate to subtropical regions.

## 3 Data Description

### 3.1 Sorghum data

**Fig. 1:**
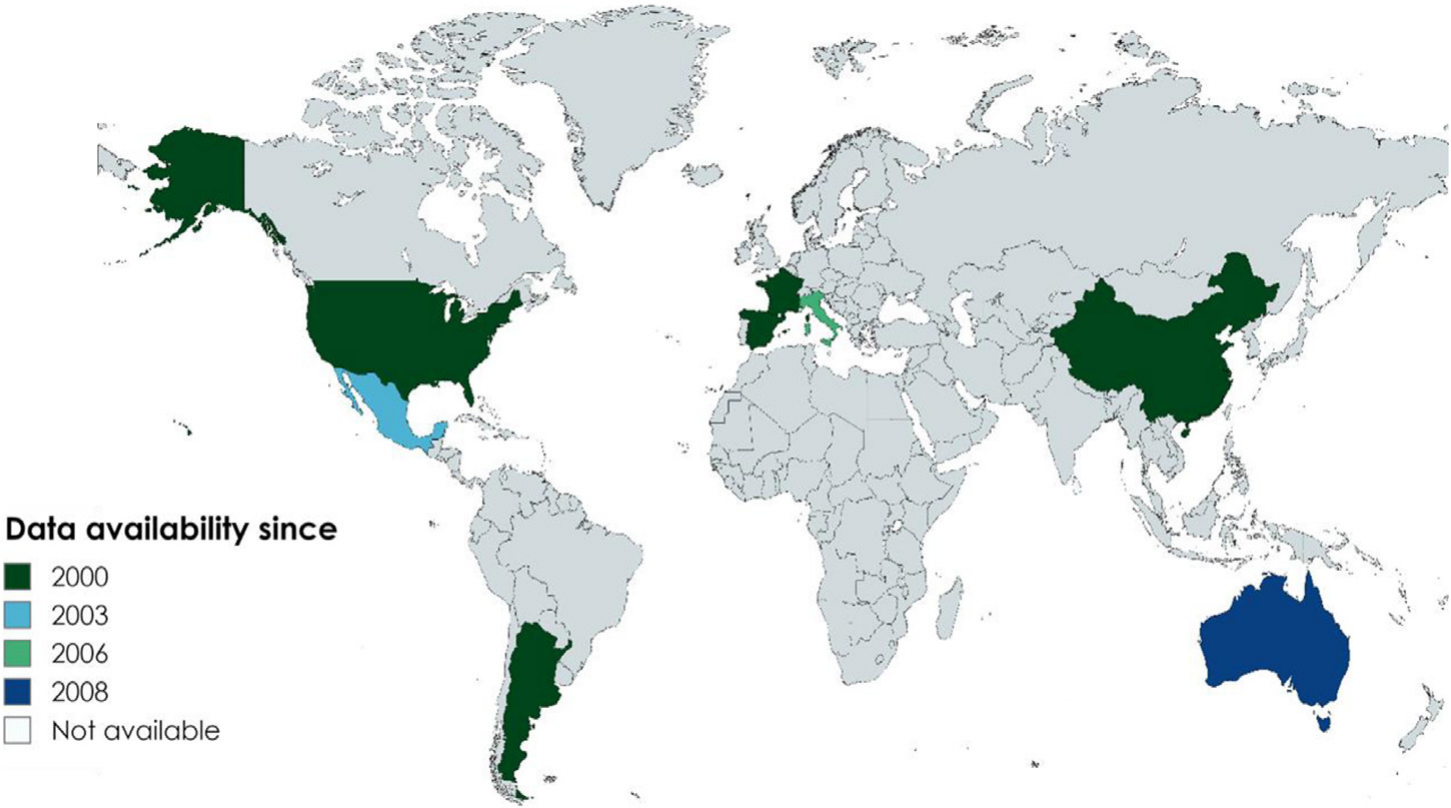
Countries included in our dataset. Each color shows how the time periods covered in the countries (last year is 2020 for all).

The dataset is available via a GitHub repository (https://github.com/mdavoudkhani/Global-sorghum-production-dataset-for-temperate-to-subtropical-regions-at-subnational-scale) containing sorghum production statistics and associated spatial data for eight countries for the period 2000–2020. The repository is structured by country, with each folder containing raw and structured data files for the periods covered by the time series, the total number of data points, the administrative levels at which the reference regions are defined, the average area of these reference regions in each country, and the URL under which the original national datasets can be found (Table 1, Fig. 1). This global dataset comprises a total of 27,222 data entries. Each entry includes the harvested area, production, and yield of grain sorghum for one specific territorial unit and year.

**Table 1:** Description of sorghum data in each country.

Country	Duration of time series	Number of data points	Administrative level	Average area (km^2^)	Reference
Argentina	2000–2020	3781	County level	4730	https://datosestimaciones.magyp.gob.ar/reportes.php?reporte=Estimaciones
Australia	2008–2020	643	Natural resource management regions	127,377	https://www.abs.gov.au/statistics/industry/agriculture/agricultural-commodities-australia
China	2000–2020	535	Provincial level	253,629	https://data.stats.gov.cn/easyquery.htm?cn=E0103
France	2000–2020	1445	Nuts3	5961	https://agreste.agriculture.gouv.fr/agreste-web/disaron/SAANR_DEVELOPPE_2/detail/
Italy	2006–2020	1098	Nuts3	2663	https://www.istat.it/en/agriculture?data-and-indicators
Mexico	2003–2020	11,007	Municipality level	1193	https://nube.siap.gob.mx/cierreagricola/
Spain	2000–2020	589	Nuts3	11,264	https://www.mapa.gob.es/es/estadistica/temas/estadisticas-agrarias/agricultura/esyrce/
The USA	2000–2020	8124	County level	2320	https://www.nass.usda.gov/Data_and_Statistics/

### 3.2 Spatial data

**Fig. 2:**
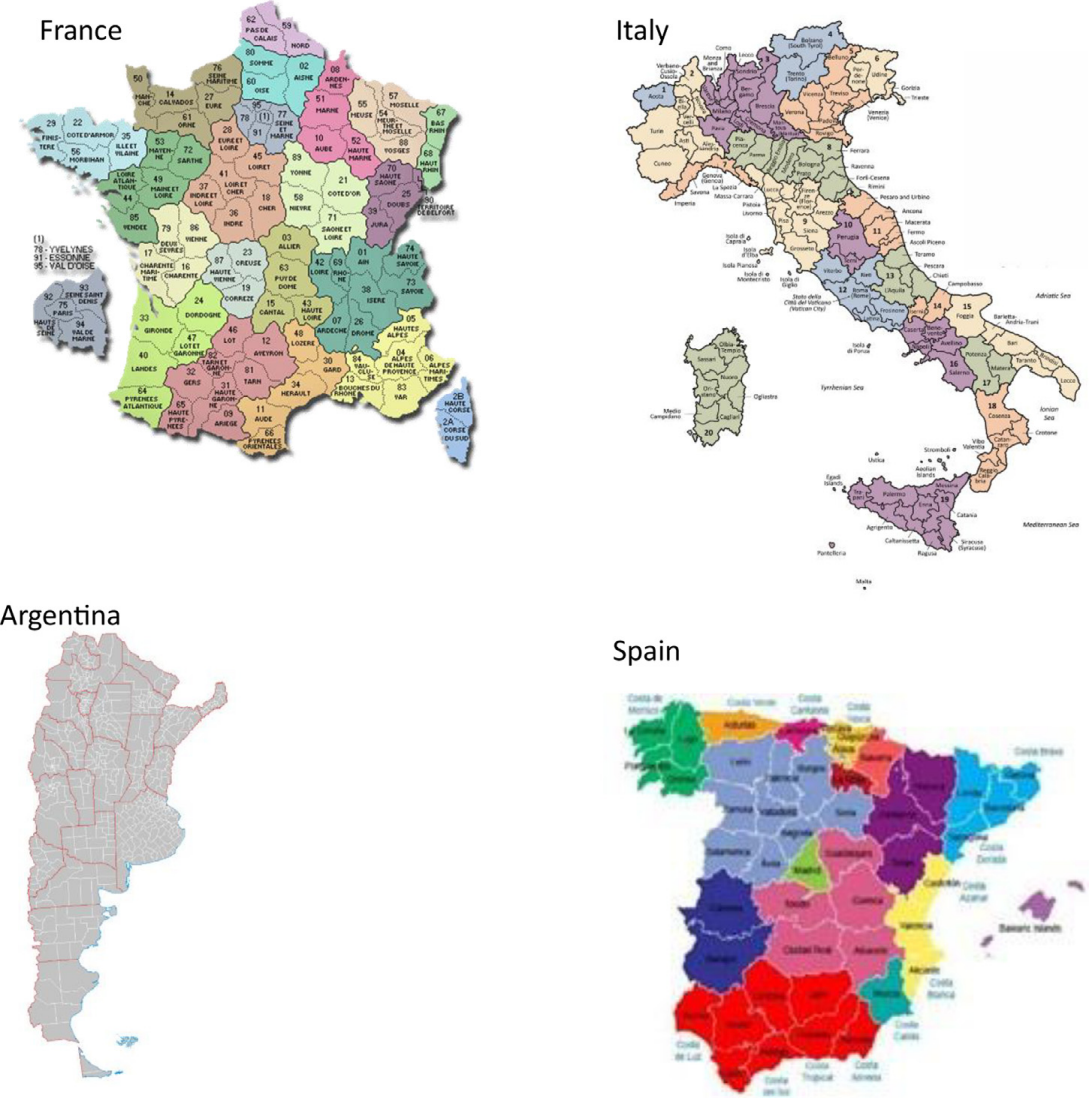

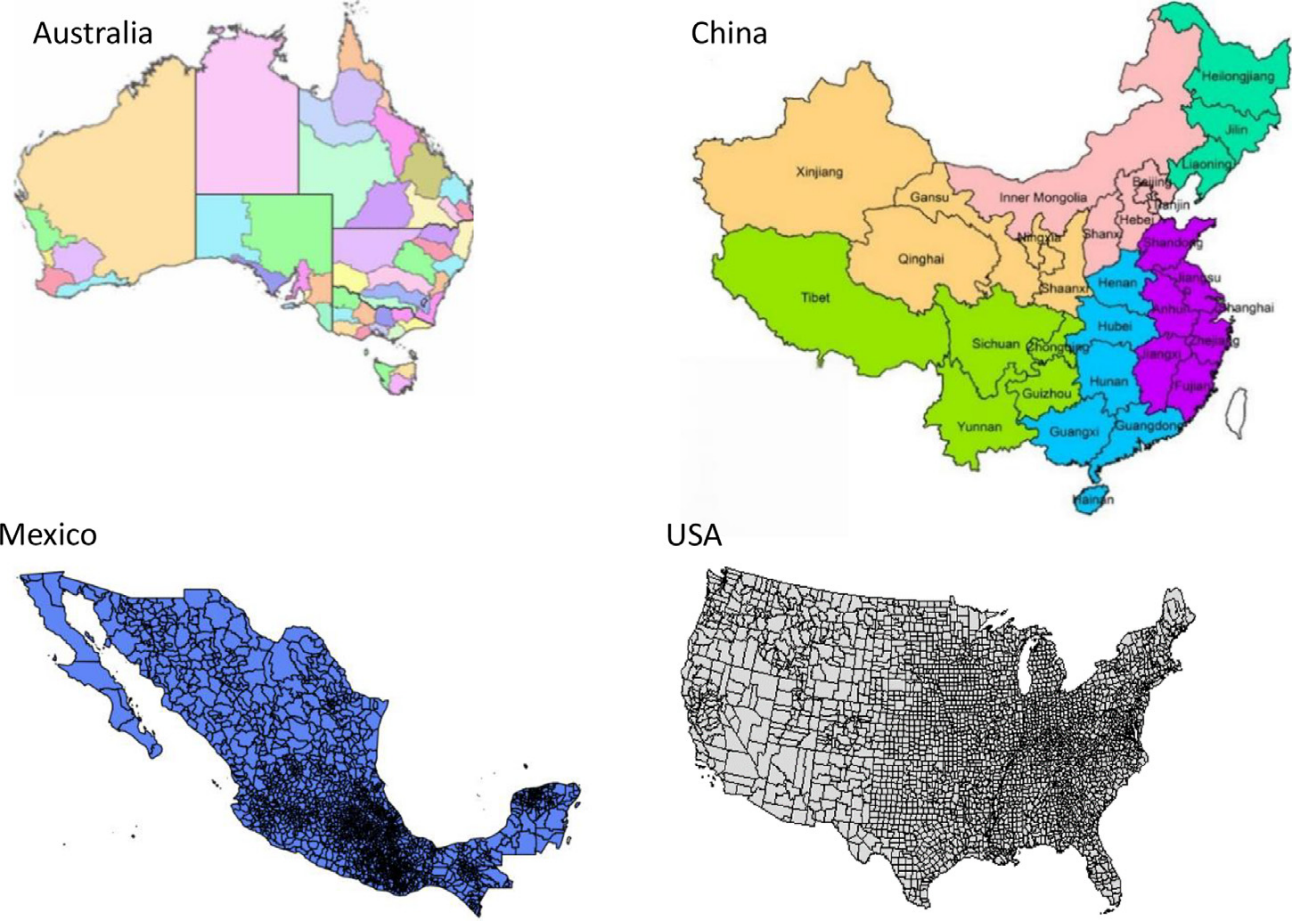
Shapefiles of administrative levels of each country in our dataset at finer subnational available. Average area (km^2^) of each administrative levels of each country: Argentina: 4730, Australia:127,377, China: 253,629, France: 5961, Italy: 2663, Mexico: 1193, Spain: 11,264, The USA: 2320.

The spatial data describing the regional units utilized in our database were sourced from various administrative subdivisions across different countries. Specifically, data from 95 departments in France, 109 departments in Italy, and 50 departments in Spain were included. In the United States and Argentina, data was collected at the county level, covering 3143 and 502 counties, respectively. In Mexico, data was recorded at the municipality level, comprising 2454 municipalities. Furthermore, in Australia, the spatial units were defined based on Natural Resource Management Regions (NRM, https://www.abs.gov.au/ausstats/abs@.nsf/Lookup/by%20Subject/1270.0.55.003∼July%202016∼Main%20Features∼Natural%20Resource%20Management%20Regions%20(NRMR)∼11) (with an average area of 127,377 km2) (NRM Regions), totaling 56 NRM regions. For China, the data was accessible at the department level, including 31 departments (Fig. 2).

### 3.3 Sorghum data visualization

**Fig. 3:**
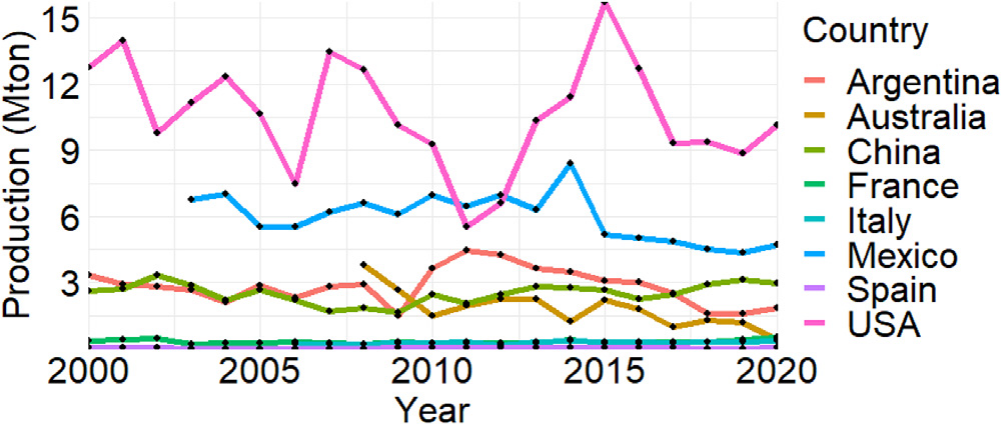
Production time series in eight selected countries. Due to data availability constraints, sorghum data for three countries starts from different years: Australia from 2008, Italy from 2006, and Mexico from 2003.

**Fig. 4:**
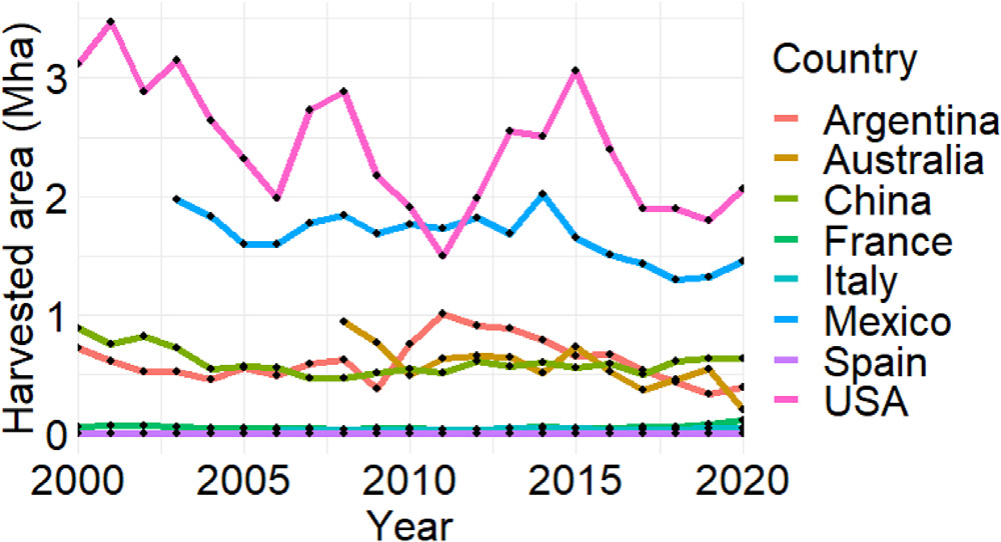
Harvested area time series in eight selected countries. Due to data availability constraints, sorghum data for three countries starts from different years: Australia from 2008, Italy from 2006, and Mexico from 2003.

**Fig. 5:**
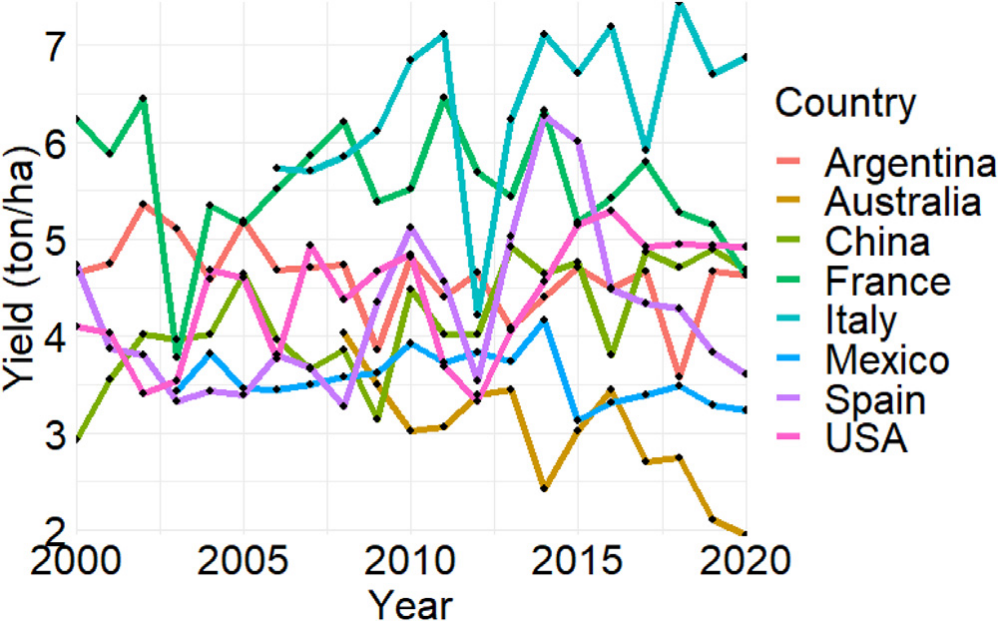
Yield time series in eight selected countries. Due to data availability constraints, sorghum data for three countries starts from different years: Australia from 2008, Italy from 2006, and Mexico from 2003.

Fig. 3, Fig. 4, Fig. 5 present the time series of sorghum production (in million tons), harvested area (in million ha), and yield (in t ha^-1^) statistics in the eight countries from 2000 to 2020, respectively. These figures summarize national-level aggregations derived from the subnational dataset.

**Fig. 6:**
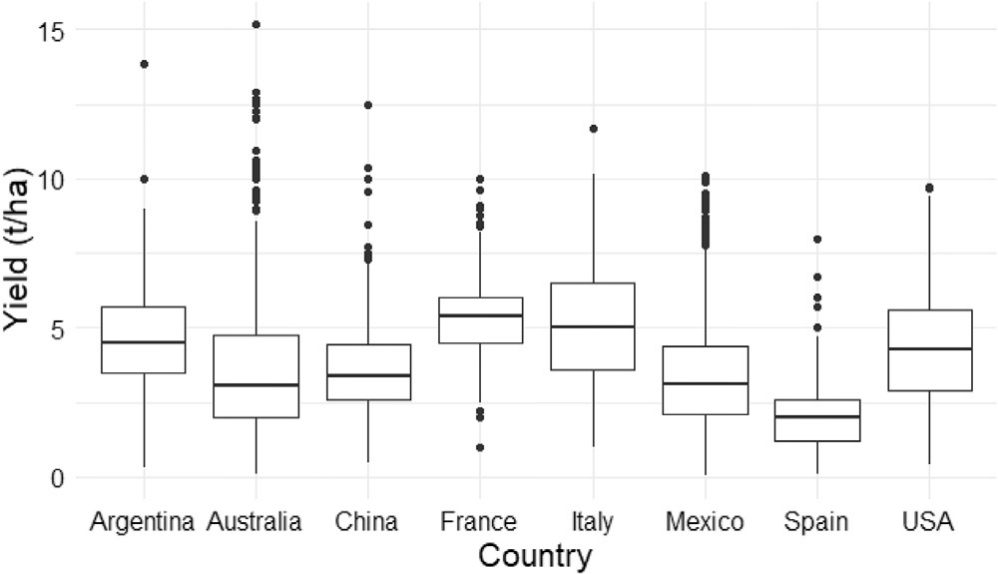
Box plots representing the variation in sorghum yields across subnational administrative levels within different countries during the years 2000–2020. Due to data availability constraints, sorghum data for three countries starts from different years: Australia from 2008, Italy from 2006, and Mexico from 2003.

**Fig. 7:**
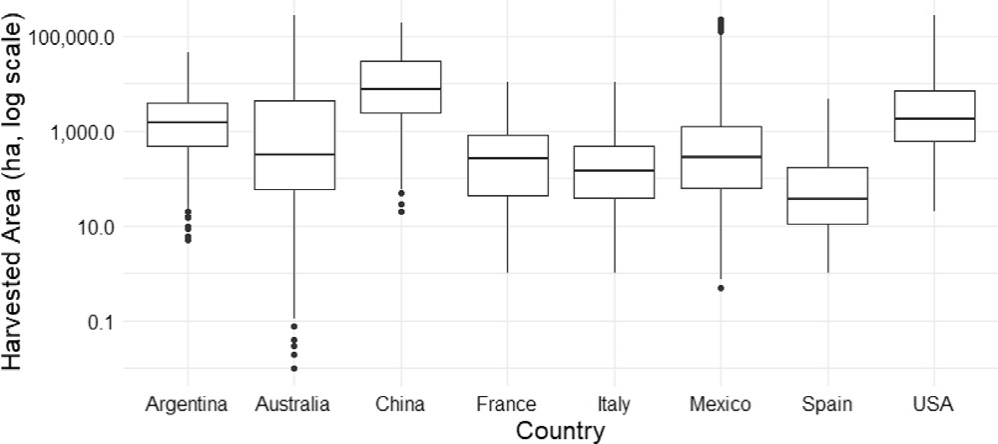
Box plots representing the variation in sorghum harvested area across subnational administrative levels within different countries during the years 2000–2020. Due to data availability constraints, sorghum data for three countries starts from different years: Australia from 2008, Italy from 2006, and Mexico from 2003.

Figs. 6 and 7 show box plots of grain sorghum yields and harvested area for grain sorghum at subnational administrative levels within different countries during the years 2000–2020, respectively.

**Fig. 8:**
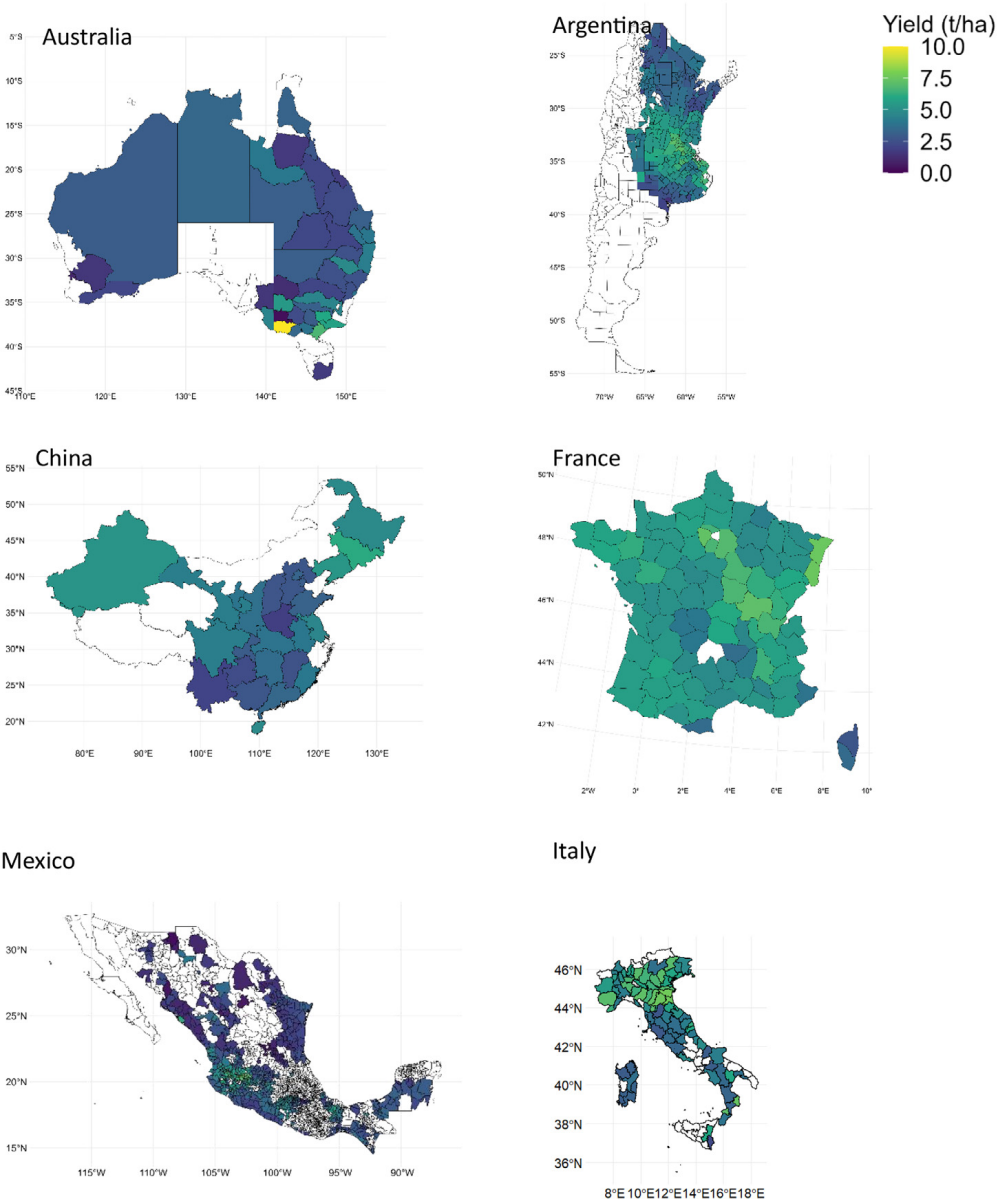

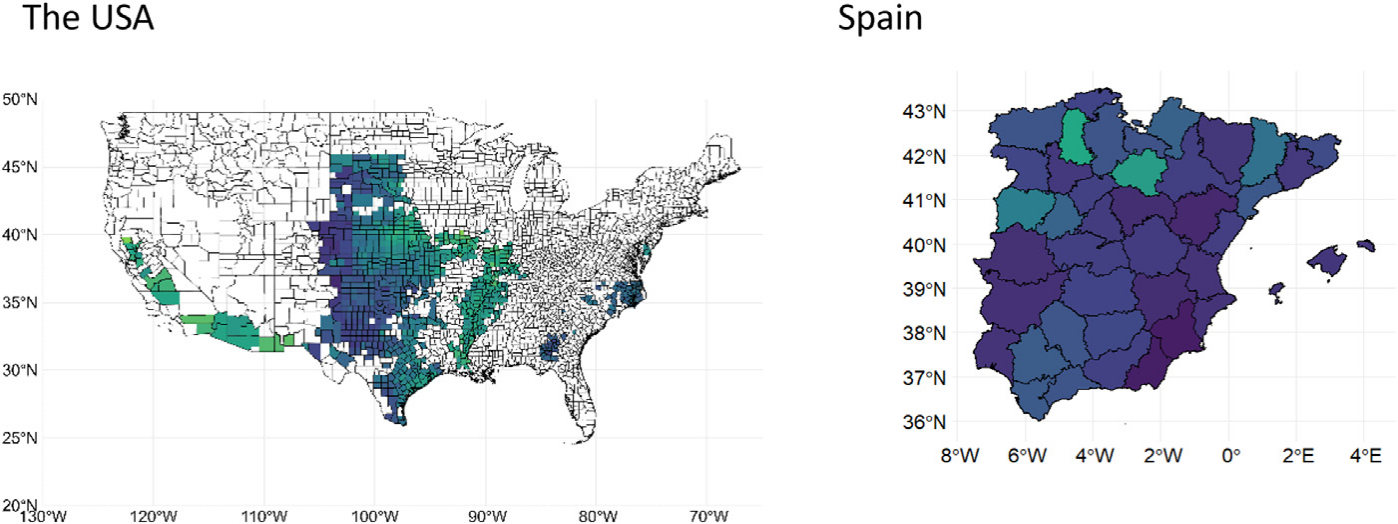
Maps of mean sorghum yield across subnational administrative levels for different countries during the years 2000–2020. Due to data availability constraints, sorghum data for three countries starts from different years: Australia from 2008, Italy from 2006, and Mexico from 2003.

**Fig. 9:**
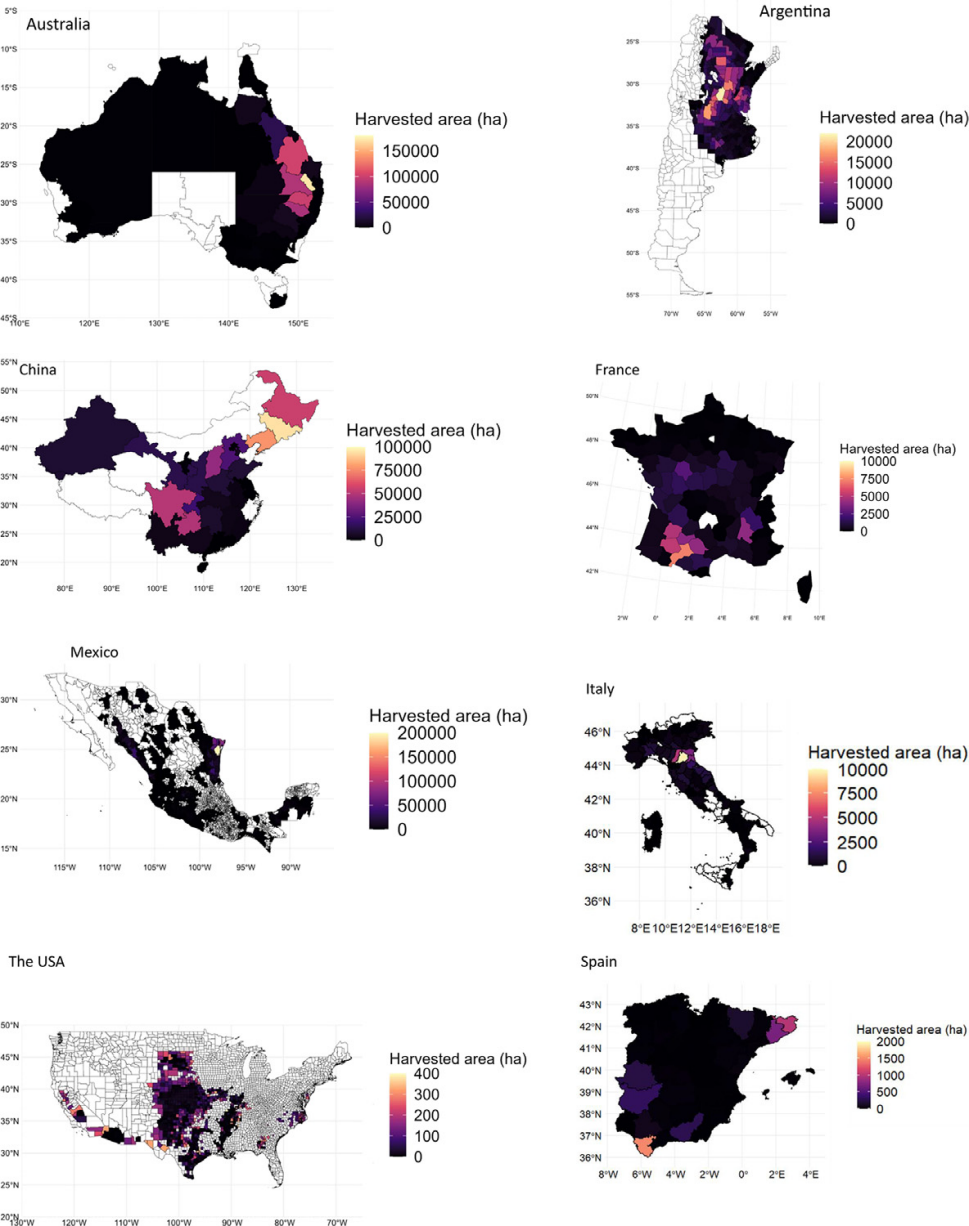
Maps of mean sorghum harvested area across subnational administrative levels for different countries during the years 2000–2020. Due to data availability constraints, sorghum data for three countries starts from different years: Australia from 2008, Italy from 2006, and Mexico from 2003.

Figs. 8 and 9 display maps of mean sorghum yield and mean sorghum harvested area across subnational administrative levels for all countries during the years 2000–2020, respectively.

**Fig. 10:**
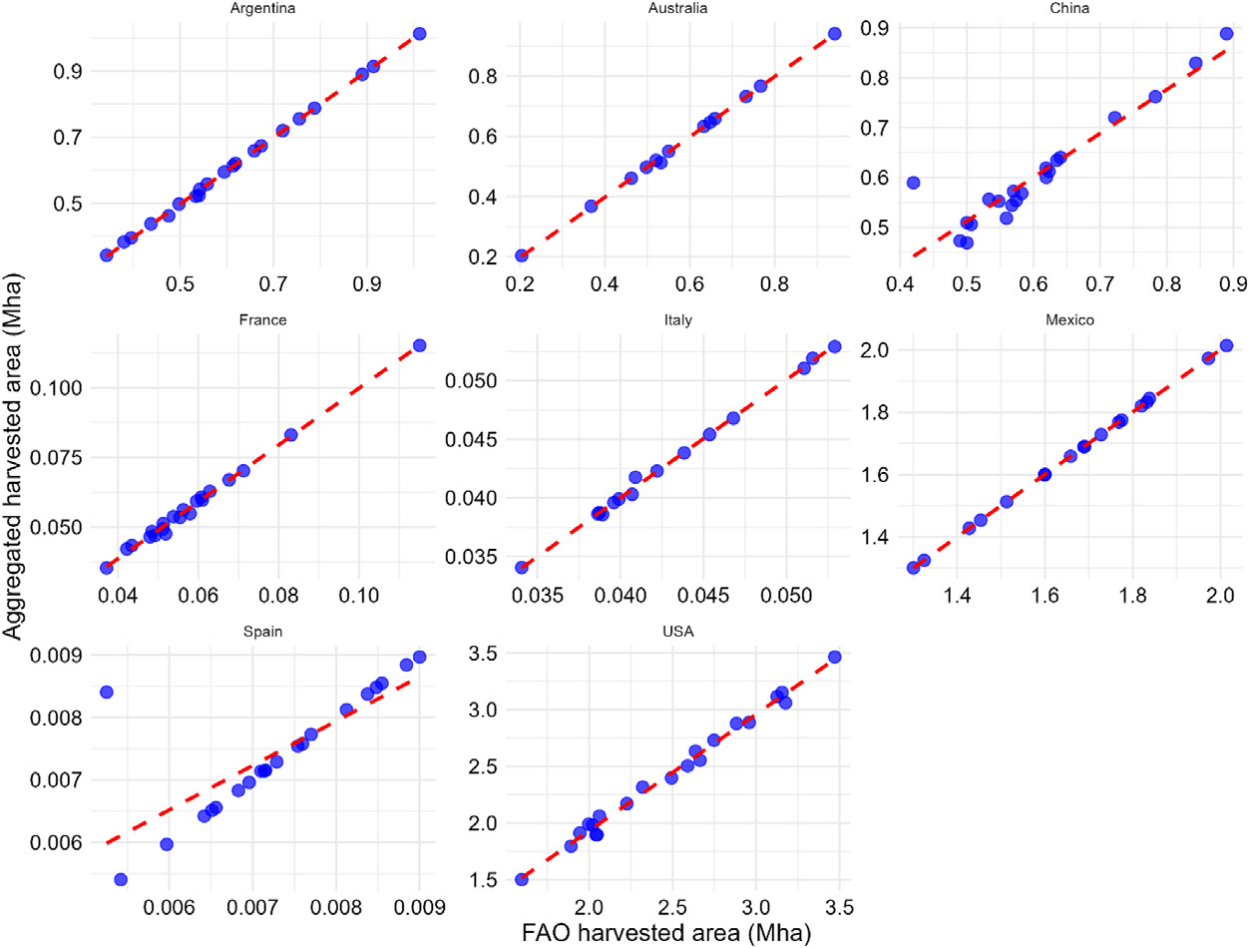
Scatter plot of harvested area values (aggregated national databases for each country vs. FAO). The dashed red line represents the *x* = *y* line. Due to data availability constraints, sorghum data for three countries starts from different years: Australia from 2008, Italy from 2006, and Mexico from 2003.

**Fig. 11:**
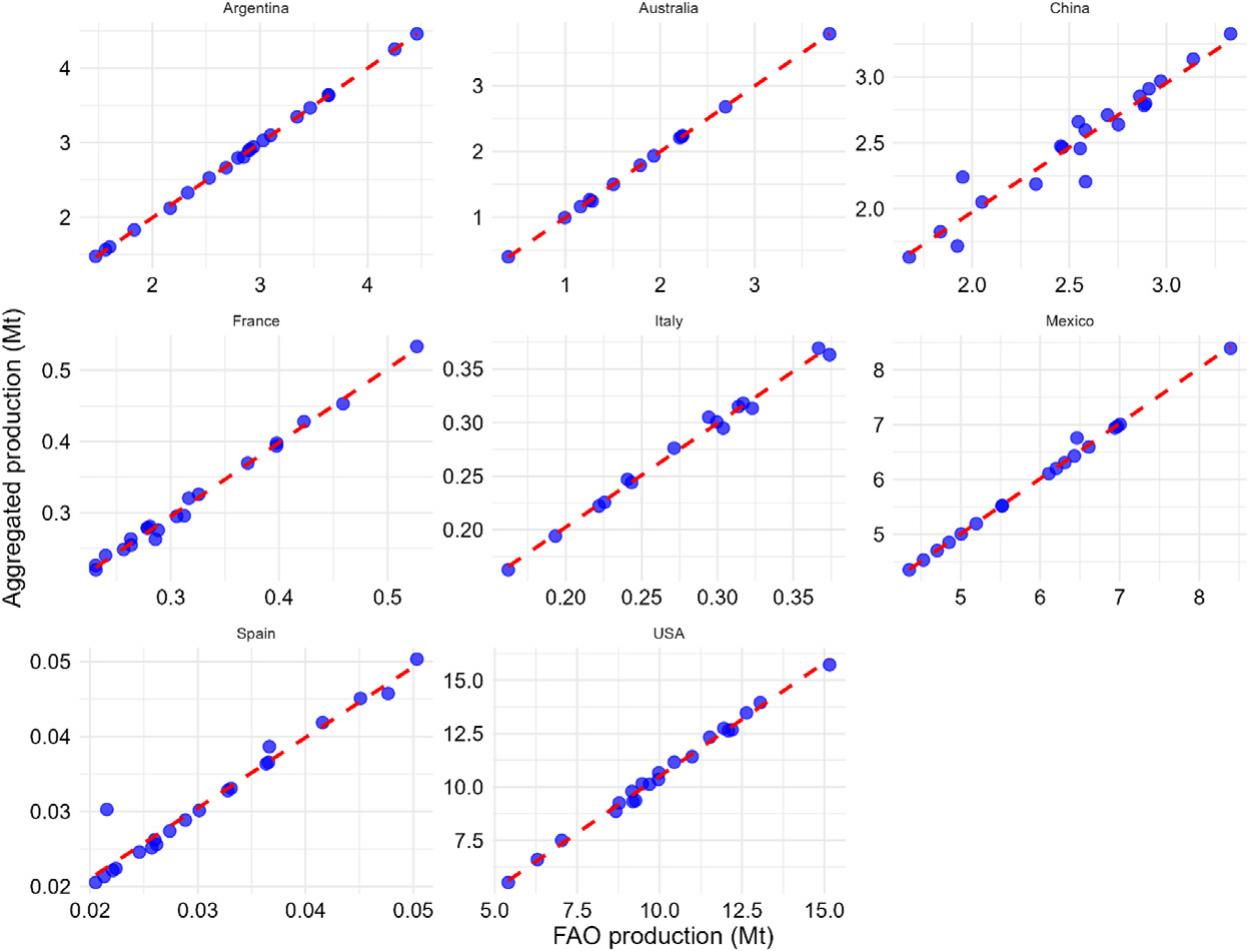
Scatter plot of production values (aggregated national databases for each country vs. FAO). The dashed red line represents the *x* = *y* line. Due to data availability constraints, sorghum data for three countries starts from different years: Australia from 2008, Italy from 2006, and Mexico from 2003.

**Fig. 12:**
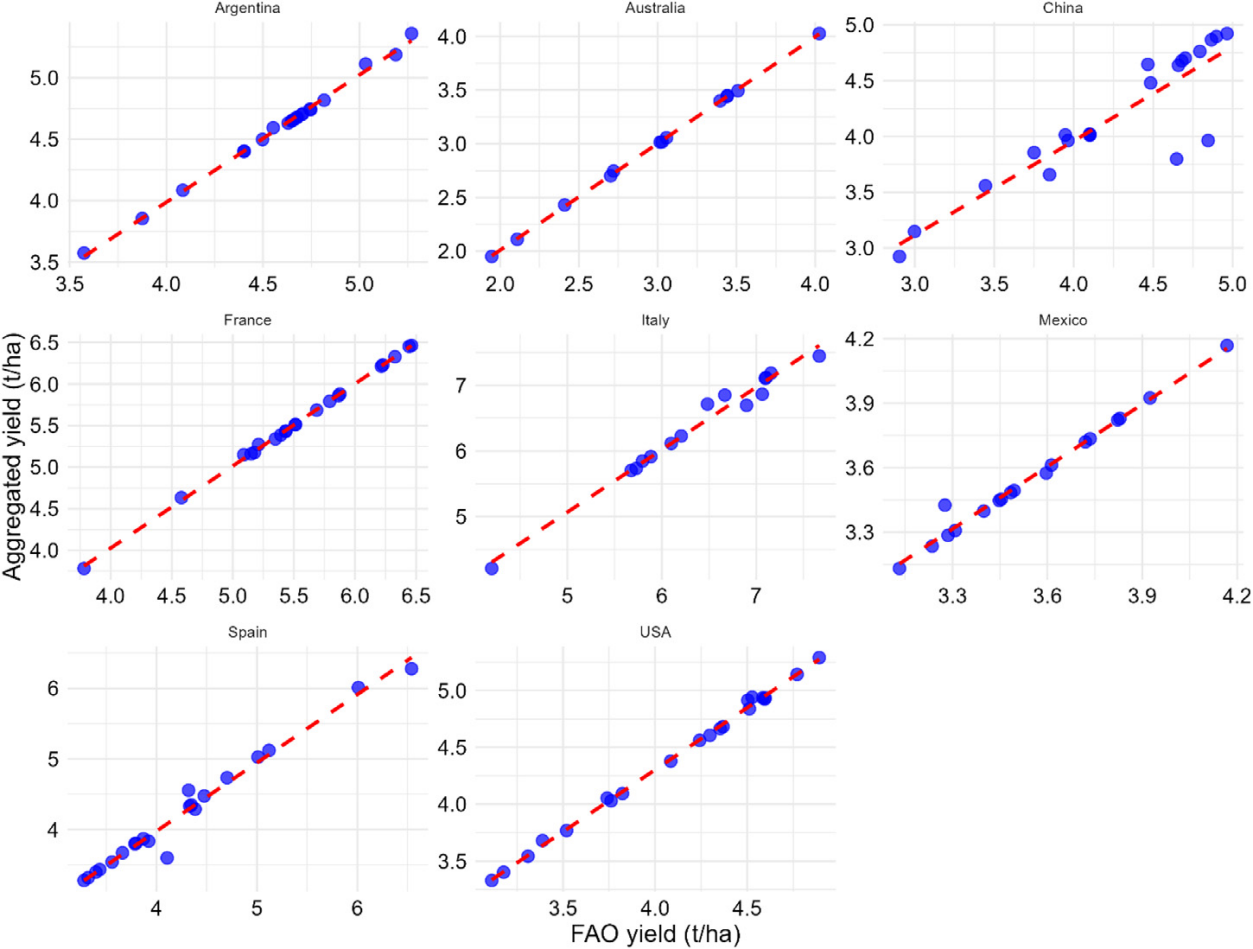
Scatter plot of yield values (aggregated national databases for each country vs. FAO). The dashed red line represents the *x* = *y* line. Due to data availability constraints, sorghum data for three countries starts from different years: Australia from 2008, Italy from 2006, and Mexico from 2003.

Table 2, Table 3, Table 4 compare Pearson correlation coefficient values, p-values, and 95 % confidence intervals for eight countries aggregated national production, harvested area, and yield statistics with the FAO dataset [2] from 2000 to 2020. Fig. 10, Fig. 11, Fig. 12 show scatter plots comparing harvested area, production values, and yield values aggregated from national databases for each country with the FAO dataset [2] from 2000 to 2020, respectively.

**Table 2:** Pearson correlation coefficient values, 95 % confidence interval, and p-value for aggregated national harvested area statistics with FAO data from 2000 to 2020.

Country	Correlation coefficient	p-value	95 percent confidence interval
Spain	0.772	0.0000	(0.510, 0.903)
The USA	0.996	0.0000	(0.989, 0.998)
Australia*	1.000	0.0000	(0.999, 1.000)
Argentina	1.000	0.0000	(0.999, 1.000)
Mexico*	1.000	0.0000	(1.000, 1.000)
China	0.936	0.0000	(0.847, 0.974)
Italy*	0.999	0.0000	(0.996, 1.000)
France	0.998	0.0000	(0.994, 0.999)

⁎ Subnational sorghum data is available from 2008 for Australia, from 2003 for Mexico, and from 2006 for Italy. Consequently, statistical analyses for these countries incorporate data starting from these respective years, while data for other countries spans the full period of 2000 to 2020.

**Table 3:** Pearson correlation coefficient values, 95 % confidence interval, and p-value for aggregated national production statistics with FAO data from 2000 to 2020.

Country	Correlation coefficient	p-value	95 percent confidence interval
Spain	0.975	0.0000	(0.938, 0.990)
The USA	0.997	0.0000	(0.992, 0.999)
Australia*	1.000	0.0000	(1.000, 1.000)
Argentina	1.000	0.0000	(1.000, 1.000)
Mexico*	0.998	0.0000	(0.994, 0.999)
China	0.961	0.0000	(0.904, 0.984)
Italy*	0.996	0.0000	(0.987, 0.999)
France	0.997	0.0000	(0.991, 0.999)

⁎ Subnational sorghum data is available from 2008 for Australia, from 2003 for Mexico, and from 2006 for Italy. Consequently, statistical analyses for these countries incorporate data starting from these respective years, while data for other countries spans the full period of 2000 to 2020.

**Table 4:** Pearson correlation coefficient values, 95 % confidence interval, and p-value for aggregated national yield statistics with FAO data from 2000 to 2020.

Country	Correlation coefficient	p-value	95 percent confidence interval
Spain	0.987	0.0000	(0.968, 0.995)
The USA	0.999	0.0000	(0.997, 1.000)
Australia*	1.000	0.0000	(0.999, 1.000)
Argentina	0.998	0.0000	(0.996, 0.999)
Mexico*	0.991	0.0000	(0.976, 0.997)
China	0.894	0.0000	(0.753, 0.957)
Italy*	0.990	0.0000	(0.968, 0.997)
France	0.999	0.0000	(0.999, 1.000)

⁎ Subnational sorghum data is available from 2008 for Australia, from 2003 for Mexico, and from 2006 for Italy. Consequently, statistical analyses for these countries incorporate data starting from these respective years, while data for other countries spans the full period of 2000 to 2020.

## 4 Experimental Design, Materials and Methods

Countries were selected based on two primary criteria. First, subnational-level sorghum data must be available for a minimum period of 10 years. Second, we selected countries within temperate to subtropical climate zones. Eight countries met these criteria: France, Italy, Spain, Argentina, Mexico, the USA, China (mainland), and Australia.

The data acquisition for this dataset involved collecting subnational-level statistics on sorghum data from publicly accessible national agricultural databases of each country. Data acquisition began with accessing each country's official agricultural statistics portal. We collected harvested area (in ha), production (in t), and yield (t ha^-1^) statistics of each country from their national agricultural statistics (Table 1). However, for three countries, sorghum data were available for shorter periods of time: Australia from 2008, Italy from 2006, and Mexico from 2003 (Table 1). Administrative units varied across countries and included departments, countries, municipalities, provinces, or natural resource regions.

An analysis was performed using national data of each country from 2000 to 2020 provided by the FAO [2] to validate our dataset. To this end, we first aggregated sorghum data at the national level. Then, we compared our dataset with the FAO dataset. For comparison, sorghum yields in our dataset were aggregated year-by-year from subnational administrative units of each country with area weighting. We used the Pearson correlation coefficient (r) to assess the correlation between these two datasets. We additionally calculated the p-value and 95 % confidence intervals for each correlation.

## Limitations

Two minor limitations were observed in the dataset. First, for China, sorghum data was available only at the provincial level rather than finer administrative units. Also, for two specific years (2006 and 2016), discrepancies in data between provincial and national totals were seen. Second, for Spain, the Spanish national dataset did not include the harvested area of the Cáceres department (2334 ha) for the year 2020, which was present in the FAO dataset.

## Ethics Statement

*N/A*.

## Credit Author Statement

**Mohsen Davoudkhani:** Conceptualization, Methodology, Formal analysis, Validation, Writing – Original Draft, Writing – Review and Editing, Visualization. **Nicolas Guilpart**: Conceptualization, Writing – Review and Editing. **David Makowski**: Conceptualization, Writing – Review and Editing. **Nicolas Viovy**: Conceptualization, Writing – Review and Editing. **Philippe Ciais**: Conceptualization, Writing – Review and Editing. **Ronny Lauerwald**: Conceptualization, Writing – Review and Editing.

## Data Availability

ZendoGlobal sorghum production dataset for temperate to subtropical regions at subnational scale over 2000–2020 (Original data).
